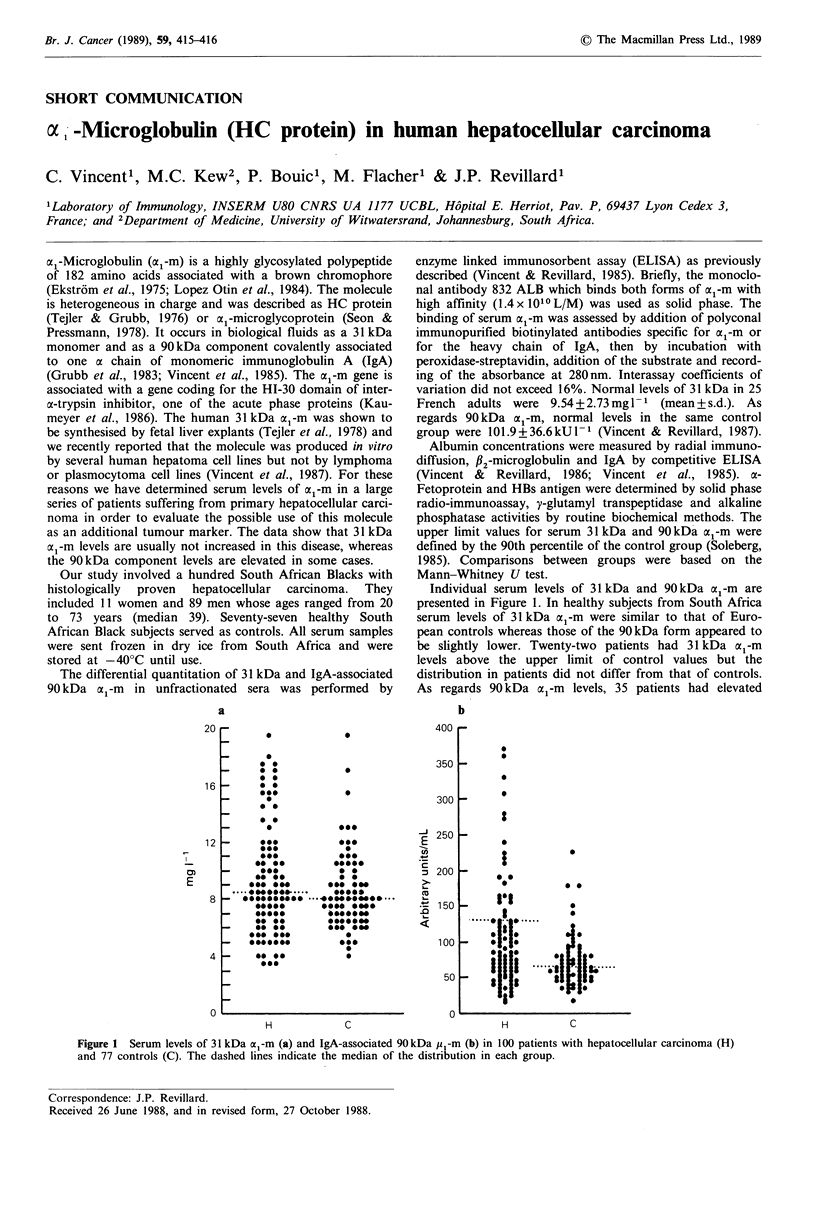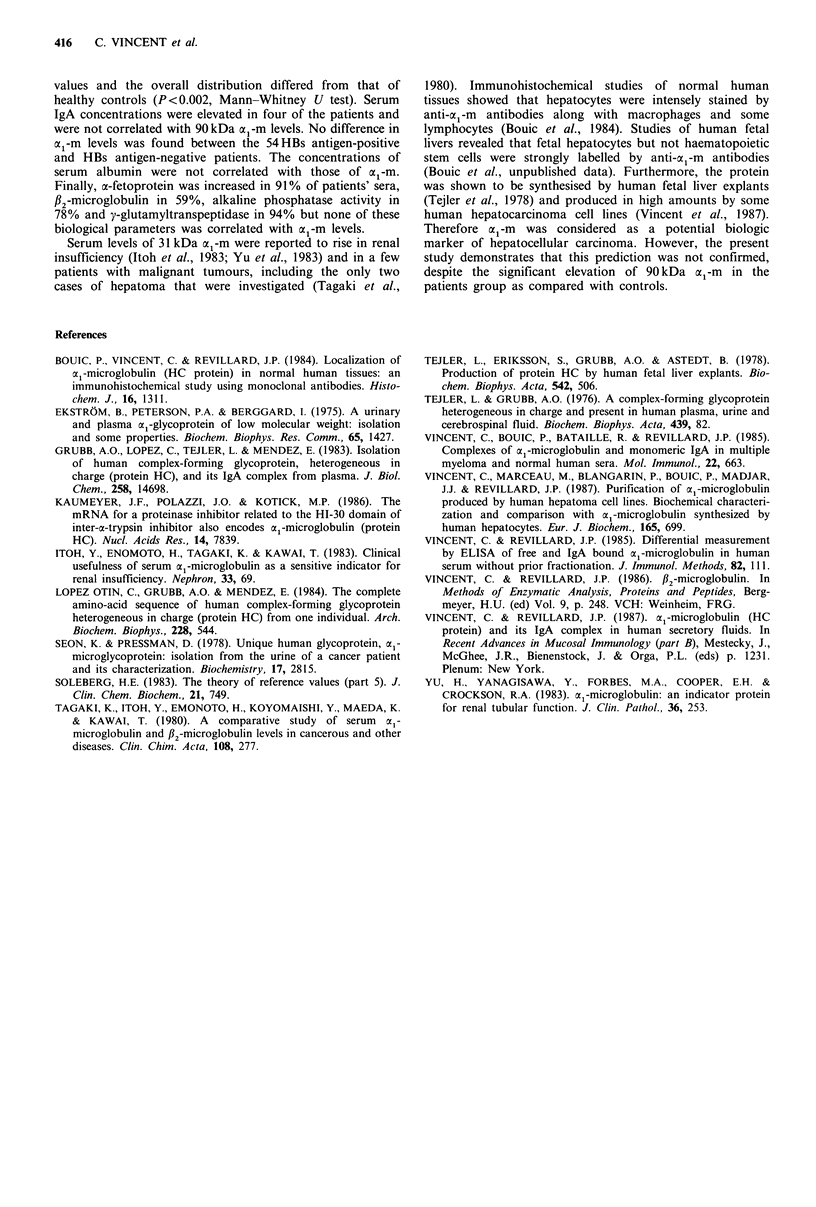# Alpha 1-microglobulin (HC protein) in human hepatocellular carcinoma.

**DOI:** 10.1038/bjc.1989.83

**Published:** 1989-03

**Authors:** C. Vincent, M. C. Kew, P. Bouic, M. Flacher, J. P. Revillard

**Affiliations:** Laboratory of Immunology, INSERM U80 CNRS UA 1177 UCBL, HÃ´pital E. Herriot, Lyon, France.


Br. J. Cancer (1989), 59, 415–416

© The Macmillan Press Ltd., 1989

SHORT COMMUNICATION

### $\alpha_1$-Microglobulin (HC protein) in human hepatocellular carcinoma

C. Vincent[1], M.C. Kew[2], P. Bouic[1], M. Flacher[1] & J.P. Revillard[1]

[1]Laboratory of Immunology, INSERM U80 CNRS UA 1177 UCBL, Hôpital E. Herriot, Pav. P, 69437 Lyon Cedex 3, France; and [2]Department of Medicine, University of Witwatersrand, Johannesburg, South Africa.

$\alpha_1$-Microglobulin ($\alpha_1$-m) is a highly glycosylated polypeptide of 182 amino acids associated with a brown chromophore (Ekström *et al.*, 1975; Lopez Otin *et al.*, 1984). The molecule is heterogeneous in charge and was described as HC protein (Tejler & Grubb, 1976) or $\alpha_1$-microglycoprotein (Seon & Pressmann, 1978). It occurs in biological fluids as a 31 kDa monomer and as a 90 kDa component covalently associated to one $\alpha$ chain of monomeric immunoglobulin A (IgA) (Grubb *et al.*, 1983; Vincent *et al.*, 1985). The $\alpha_1$-m gene is associated with a gene coding for the HI-30 domain of inter-$\alpha$-trypsin inhibitor, one of the acute phase proteins (Kaumeyer *et al.*, 1986). The human 31 kDa $\alpha_1$-m was shown to be synthesised by fetal liver explants (Tejler *et al.*, 1978) and we recently reported that the molecule was produced *in vitro* by several human hepatoma cell lines but not by lymphoma or plasmocytoma cell lines (Vincent *et al.*, 1987). For these reasons we have determined serum levels of $\alpha_1$-m in a large series of patients suffering from primary hepatocellular carcinoma in order to evaluate the possible use of this molecule as an additional tumour marker. The data show that 31 kDa $\alpha_1$-m levels are usually not increased in this disease, whereas the 90 kDa component levels are elevated in some cases.

Our study involved a hundred South African Blacks with histologically proven hepatocellular carcinoma. They included 11 women and 89 men whose ages ranged from 20 to 73 years (median 39). Seventy-seven healthy South African Black subjects served as controls. All serum samples were sent frozen in dry ice from South Africa and were stored at −40°C until use.

The differential quantitation of 31 kDa and IgA-associated 90 kDa $\alpha_1$-m in unfractionated sera was performed by enzyme linked immunosorbent assay (ELISA) as previously described (Vincent & Revillard, 1985). Briefly, the monoclonal antibody 832 ALB which binds both forms of $\alpha_1$-m with high affinity ($1.4 \times 10^{10}$ L/M) was used as solid phase. The binding of serum $\alpha_1$-m was assessed by addition of polyconal immunopurified biotinylated antibodies specific for $\alpha_1$-m or for the heavy chain of IgA, then by incubation with peroxidase-streptavidin, addition of the substrate and recording of the absorbance at 280 nm. Interassay coefficients of variation did not exceed 16%. Normal levels of 31 kDa in 25 French adults were $9.54 \pm 2.73\,\mathrm{mg\,l^{-1}}$ (mean ± s.d.). As regards 90 kDa $\alpha_1$-m, normal levels in the same control group were $101.9 \pm 36.6\,\mathrm{kU\,l^{-1}}$ (Vincent & Revillard, 1987).

Albumin concentrations were measured by radial immunodiffusion, $\beta_2$-microglobulin and IgA by competitive ELISA (Vincent & Revillard, 1986; Vincent *et al.*, 1985). $\alpha$-Fetoprotein and HBs antigen were determined by solid phase radio-immunoassay, $\gamma$-glutamyl transpeptidase and alkaline phosphatase activities by routine biochemical methods. The upper limit values for serum 31 kDa and 90 kDa $\alpha_1$-m were defined by the 90th percentile of the control group (Soleberg, 1985). Comparisons between groups were based on the Mann–Whitney *U* test.

Individual serum levels of 31 kDa and 90 kDa $\alpha_1$-m are presented in Figure 1. In healthy subjects from South Africa serum levels of 31 kDa $\alpha_1$-m were similar to that of European controls whereas those of the 90 kDa form appeared to be slightly lower. Twenty-two patients had 31 kDa $\alpha_1$-m levels above the upper limit of control values but the distribution in patients did not differ from that of controls. As regards 90 kDa $\alpha_1$-m levels, 35 patients had elevated

**Figure 1** Serum levels of 31 kDa $\alpha_1$-m (**a**) and IgA-associated 90 kDa $\mu_1$-m (**b**) in 100 patients with hepatocellular carcinoma (H) and 77 controls (C). The dashed lines indicate the median of the distribution in each group.

Correspondence: J.P. Revillard.

Received 26 June 1988, and in revised form, 27 October 1988.

values and the overall distribution differed from that of healthy controls ($P<0.002$, Mann–Whitney $U$ test). Serum IgA concentrations were elevated in four of the patients and were not correlated with 90 kDa $\alpha_1$-m levels. No difference in $\alpha_1$-m levels was found between the 54 HBs antigen-positive and HBs antigen-negative patients. The concentrations of serum albumin were not correlated with those of $\alpha_1$-m. Finally, $\alpha$-fetoprotein was increased in 91% of patients' sera, $\beta_2$-microglobulin in 59%, alkaline phosphatase activity in 78% and $\gamma$-glutamyltranspeptidase in 94% but none of these biological parameters was correlated with $\alpha_1$-m levels.

Serum levels of 31 kDa $\alpha_1$-m were reported to rise in renal insufficiency (Itoh *et al.*, 1983; Yu *et al.*, 1983) and in a few patients with malignant tumours, including the only two cases of hepatoma that were investigated (Tagaki *et al.*, 1980). Immunohistochemical studies of normal human tissues showed that hepatocytes were intensely stained by anti-$\alpha_1$-m antibodies along with macrophages and some lymphocytes (Bouic *et al.*, 1984). Studies of human fetal livers revealed that fetal hepatocytes but not haematopoietic stem cells were strongly labelled by anti-$\alpha_1$-m antibodies (Bouic *et al.*, unpublished data). Furthermore, the protein was shown to be synthesised by human fetal liver explants (Tejler *et al.*, 1978) and produced in high amounts by some human hepatocarcinoma cell lines (Vincent *et al.*, 1987). Therefore $\alpha_1$-m was considered as a potential biologic marker of hepatocellular carcinoma. However, the present study demonstrates that this prediction was not confirmed, despite the significant elevation of 90 kDa $\alpha_1$-m in the patients group as compared with controls.

### References

BOUIC, P., VINCENT, C. & REVILLARD, J.P. (1984). Localization of $\alpha_1$-microglobulin (HC protein) in normal human tissues: an immunohistochemical study using monoclonal antibodies. *Histochem. J.*, **16**, 1311.

EKSTRÖM, B., PETERSON, P.A. & BERGGARD, I. (1975). A urinary and plasma $\alpha_1$-glycoprotein of low molecular weight: isolation and some properties. *Biochem. Biophys. Res. Comm.*, **65**, 1427.

GRUBB, A.O., LOPEZ, C., TEJLER, L. & MENDEZ, E. (1983). Isolation of human complex-forming glycoprotein, heterogeneous in charge (protein HC), and its IgA complex from plasma. *J. Biol. Chem.*, **258**, 14698.

KAUMEYER, J.F., POLAZZI, J.O. & KOTICK, M.P. (1986). The mRNA for a proteinase inhibitor related to the HI-30 domain of inter-$\alpha$-trypsin inhibitor also encodes $\alpha_1$-microglobulin (protein HC). *Nucl. Acids Res.*, **14**, 7839.

ITOH, Y., ENOMOTO, H., TAGAKI, K. & KAWAI, T. (1983). Clinical usefulness of serum $\alpha_1$-microglobulin as a sensitive indicator for renal insufficiency. *Nephron*, **33**, 69.

LOPEZ OTIN, C., GRUBB, A.O. & MENDEZ, E. (1984). The complete amino-acid sequence of human complex-forming glycoprotein heterogeneous in charge (protein HC) from one individual. *Arch. Biochem. Biophys.*, **228**, 544.

SEON, K. & PRESSMAN, D. (1978). Unique human glycoprotein, $\alpha_1$-microglycoprotein: isolation from the urine of a cancer patient and its characterization. *Biochemistry*, **17**, 2815.

SOLEBERG, H.E. (1983). The theory of reference values (part 5). *J. Clin. Chem. Biochem.*, **21**, 749.

TAGAKI, K., ITOH, Y., EMONOTO, H., KOYOMAISHI, Y., MAEDA, K. & KAWAI, T. (1980). A comparative study of serum $\alpha_1$-microglobulin and $\beta_2$-microglobulin levels in cancerous and other diseases. *Clin. Chim. Acta*, **108**, 277.

TEJLER, L., ERIKSSON, S., GRUBB, A.O. & ASTEDT, B. (1978). Production of protein HC by human fetal liver explants. *Biochem. Biophys. Acta*, **542**, 506.

TEJLER, L. & GRUBB, A.O. (1976). A complex-forming glycoprotein heterogeneous in charge and present in human plasma, urine and cerebrospinal fluid. *Biochem. Biophys. Acta*, **439**, 82.

VINCENT, C., BOUIC, P., BATAILLE, R. & REVILLARD, J.P. (1985). Complexes of $\alpha_1$-microglobulin and monomeric IgA in multiple myeloma and normal human sera. *Mol. Immunol.*, **22**, 663.

VINCENT, C., MARCEAU, M., BLANGARIN, P., BOUIC, P., MADJAR, J.J. & REVILLARD, J.P. (1987). Purification of $\alpha_1$-microglobulin produced by human hepatoma cell lines. Biochemical characterization and comparison with $\alpha_1$-microglobulin synthesized by human hepatocytes. *Eur. J. Biochem.*, **165**, 699.

VINCENT, C. & REVILLARD, J.P. (1985). Differential measurement by ELISA of free and IgA bound $\alpha_1$-microglobulin in human serum without prior fractionation. *J. Immunol. Methods*, **82**, 111.

VINCENT, C. & REVILLARD, J.P. (1986). $\beta_2$-microglobulin. In *Methods of Enzymatic Analysis, Proteins and Peptides*, Bergmeyer, H.U. (ed) Vol. 9, p. 248. VCH: Weinheim, FRG.

VINCENT, C. & REVILLARD, J.P. (1987). $\alpha_1$-microglobulin (HC protein) and its IgA complex in human secretory fluids. In *Recent Advances in Mucosal Immunology* (*part B*), Mestecky, J., McGhee, J.R., Bienenstock, J. & Orga, P.L. (eds) p. 1231. Plenum: New York.

YU, H., YANAGISAWA, Y., FORBES, M.A., COOPER, E.H. & CROCKSON, R.A. (1983). $\alpha_1$-microglobulin: an indicator protein for renal tubular function. *J. Clin. Pathol.*, **36**, 253.